# Enhanced-RICAP: a novel data augmentation strategy for improved deep learning-based plant disease identification and mobile diagnosis

**DOI:** 10.3389/fpls.2025.1646611

**Published:** 2025-09-24

**Authors:** Mamadou Bailo Diallo, Yue Li, Okafor Sylevester Chukwuka, Solomon Boamah, Yuhong Gao, Mohamed Meyer Kana Kone, Gelebo Rocho, Linjing Wei

**Affiliations:** ^1^ College of Information Sciences and Technology, Gansu Agricultural University, Lanzhou, China; ^2^ Gansu Provincial Key Laboratory of Aridland Crop Science, Gansu Agricultural University, Lanzhou, China; ^3^ College of Plant Protection, Gansu Agricultural University, Lanzhou, China; ^4^ College of Agronomy, Gansu Agricultural University, Lanzhou, China; ^5^ Department of Computer Sciences and Technology, Nanchang University, Nanchang, Jiangxi, China; ^6^ College of Food Science and Engineering, Gansu Agricultural University, Lanzhou, China

**Keywords:** deep learning, plant disease identification, data augmentation, food security, sustainable agriculture

## Abstract

**Introduction:**

Plant diseases pose a significant threat to global food security and agricultural productivity, making accurate and timely disease identification essential for effective crop management and minimizing economic losses. Although data augmentation techniques such as RICAP improve model robustness, their reliance on randomly extracted image regions can introduce label noise, potentially misleading the training of deep learning models.

**Methods:**

This study introduces Enhanced-RICAP, an advanced data augmentation technique designed to improve the accuracy of deep learning models for plant disease detection. Enhanced-RICAP replaces random patch selection with an attention module guided by class activation maps, focusing on discriminative regions, Enhanced-RICAP reduces label noise and improves model accuracy for plant disease detection, addressing a key limitation of traditional augmentation methods. The method was evaluated using several deep learning architectures, such as ResNet18, ResNet34, ResNet50, EfficientNet-b, and Xception, on the cassava leaf disease and PlantVillage tomato leaf disease datasets.

**Results:**

The experimental results demonstrate that Enhanced-RICAP consistently outperforms existing augmentation methods, including CutMix, MixUp, CutOut, Hide-and-Seek, and RICAP, across key evaluation metrics: accuracy, precision, recall, and F1-score. The ResNet18+Enhanced-RICAP configuration achieved 99.86% accuracy on the tomato leaf disease dataset, whereas the Xception+Enhanced-RICAP model attained 96.64% accuracy in classifying four cassava leaf disease categories.

**Discussion and Conclusion:**

To bridge the gap between research and practical application, the ResNet18+Enhanced-RICAP model was deployed in PlantDisease, a mobile application that enables real-time disease identification and management recommendations. This approach supports sustainable agriculture and strengthens food security by providing farmers with accessible and reliable diagnostic tools.

## 1 Introduction

Agriculture remains the primary source of livelihood for a large portion of the global population. However, food security continues to be threatened by a range of factors, including climate change and plant diseases (Khan et al., 2022). Plant diseases are not only a global threat to food security but also pose devastating risks to smallholder farmers whose livelihoods depend on healthy crops. The rapid commercialization of agricultural (Abbas et al., 2021) practices has further impacted the environment, complicating efforts to maintain sustainable farming. Among the critical challenges in modern agriculture is the early and accurate identification of plant diseases, such as cassava leaf and tomato leaf diseases. Timely identification of plant diseases is essential to prevent the spread of infections to healthy plants, thereby reducing the risk of substantial economic losses. The consequences of plant diseases can range from minor symptoms to the complete destruction of plantations, severely undermining agricultural productivity and economic stability. In particular, the increased cultivation of cassava and tomato crops has made disease identification increasingly important. These crops are susceptible to various infections, which often present with subtle and overlapping symptoms that complicate visual diagnosis. Although expert-based manual inspection remains a primary method for diagnosing plant diseases, its dependence on human judgment introduces variability and inefficiency, often leading to delayed or inaccurate assessments (Zarboubi et al., 2025). These diagnostic challenges, coupled with environmental influences, contribute to delayed or ineffective treatment, reducing yield and crop quality. To address these limitations, the integration of computer vision and deep learning offers a promising solution for developing automated and scalable plant disease identification systems. Such tools can support farmers in early diagnosis and more effective disease management, ultimately strengthening food security and agricultural resilience.

Recent advances in deep learning, particularly Convolutional Neural Networks (CNNs), have shown great potential in addressing challenges in plant disease identification by automating the feature extraction and identification process, enhancing the reliability and efficiency of the identification (Jafar et al., 2024; Sajitha et al., 2024). CNNs are widely used in diverse domains such as medical imaging, object detection, and agricultural disease diagnosis, due to their capacity to automatically learn and capture meaningful features from images (Li et al., 2024; Zhang and Mu, 2024). Several studies have employed CNN-based approaches to identify diseases in tomato and cassava leaves. For tomato disease identification, researchers have developed custom CNN architectures capable of distinguishing between multiple disease types and localizing affected regions on the leaf surface (Agarwal et al., 2020; Zhang et al., 2020). Popular CNN architectures such as VGGNet, RNeT, GoogLeNet, MobileNet, and Inception have been adapted for plant disease identification in numerous studies (Sanida et al., 2023; Vengaiah and Priyadharshini, 2023; Ajitha et al., 2024). In relation to cassava disease identification, CNN-based methods have also gained traction. Ayu et al. (2021) proposed a customized version of MobileNetV2 for detecting cassava leaf diseases, while Singh et al. (2023) explored the application of InceptionRNeTV2 to improve disease identification. Sholihin et al. (2023) utilized AlexNet as a feature extractor in combination with a support vector machine (SVM) classifier. Additionally, pre-trained CNN models, such as VGG19, have been employed using transfer learning techniques to enhance model generalization and reduce the need for extensive training from scratch (Alford and Tuba, 2024).

A variety of data augmentation techniques (Zhang et al., 2018; Summers and Dinneen, 2019) aim at mixing data to enhance data diversity. As a result, the data mixing strategy compels the neural network to attend to multiple objects and regions in the input image, thereby enhancing its feature extraction capabilities for the networks. Among data augmentation techniques, CutOut (DeVries, 2017) exemplifies a method that enhances training data by systematically removing rectangular regions from images. Another category comprises data-mixing techniques (Inoue, 2018; Tokozume et al., 2018), which have garnered significant attention in the domain of image identification in recent years. Mixing data to extend the training distribution was first proposed by Zhang et al (Zhang et al., 2018). MixUp entails generating training samples by linearly mixing images and fusing their labels using the same coefficients. This technique has demonstrated notable effectiveness in mitigating the impact of noisy labels and enhancing overall model performance.

Recently, Mixup variants (Takahashi et al., 2018; Guo et al., 2019; Summers and Dinneen, 2019) have been proposed; they perform feature-level interpolation and other types of transformations. Random Image Cropping and Patching (RICAP) (Takahashi et al., 2018) is introduced as a data augmentation method that enhances training diversity by cropping regions from four distinct images and combining them into a single composite image, unlike traditional approaches that utilize only two images. However, the risk of label noise increases since the region randomly extracted to form the mixed image may cover a meaningless region of their respective source image. CutMix, introduced by Yun et al (Yun et al., 2019), generates new images by replacing a region of one image with a patch from another. The corresponding labels are combined in proportion to the area of the exchanged patches, similar to the approach used in MixUp. Based on CutMix, and SaliencyMix (Uddin et al., 2020) guide mixing patches by saliency regions in the image (based on CAM or a saliency detector) to obtain mixed samples with more class-relevant information; ResizeMix (Qin et al., 2020) maintains the information integrity by replacing one resized image directly into a rectangular area of another image. Despite their contributions, these previous studies often overlook thorough evaluations, particularly with respect to localization performance and the ability to capture discriminative regions.

The objective of this study is to create a more efficient data-mixing augmentation technique for enhancing the identification of cassava and tomato leaf diseases. Unlike the original RICAP (Takahashi et al., 2018) which relies on random region box generation, Enhanced-RICAP incorporates an attention module. Specifically, it leverages class activation maps to extract discriminative regions from four distinct images, which are then patched together to match the size of the original image. The corresponding labels are mixed according to the semantic composition of the newly generated image. This approach improves the model’s ability to generalize and reliably detect plant diseases while reducing the risk of overfitting.

- We introduce a data-mixing augmentation technique that efficiently preserves important discriminative regions while introducing sufficient variability.
- Applying CAM to guide the augmentation process, ensuring that crucial features are not obscured.
- We provide a comparative analysis demonstrating that Enhanced-RICAP out-performs existing augmentation methods on both cassava and tomato dataset, and deploys the resulting model in a mobile app for real-time, on-site disease identification reducing reliance on experts and enabling more timely, accurate crop management.

## 2 Materials and methods

### 2.1 Dataset

**Figure 1:**
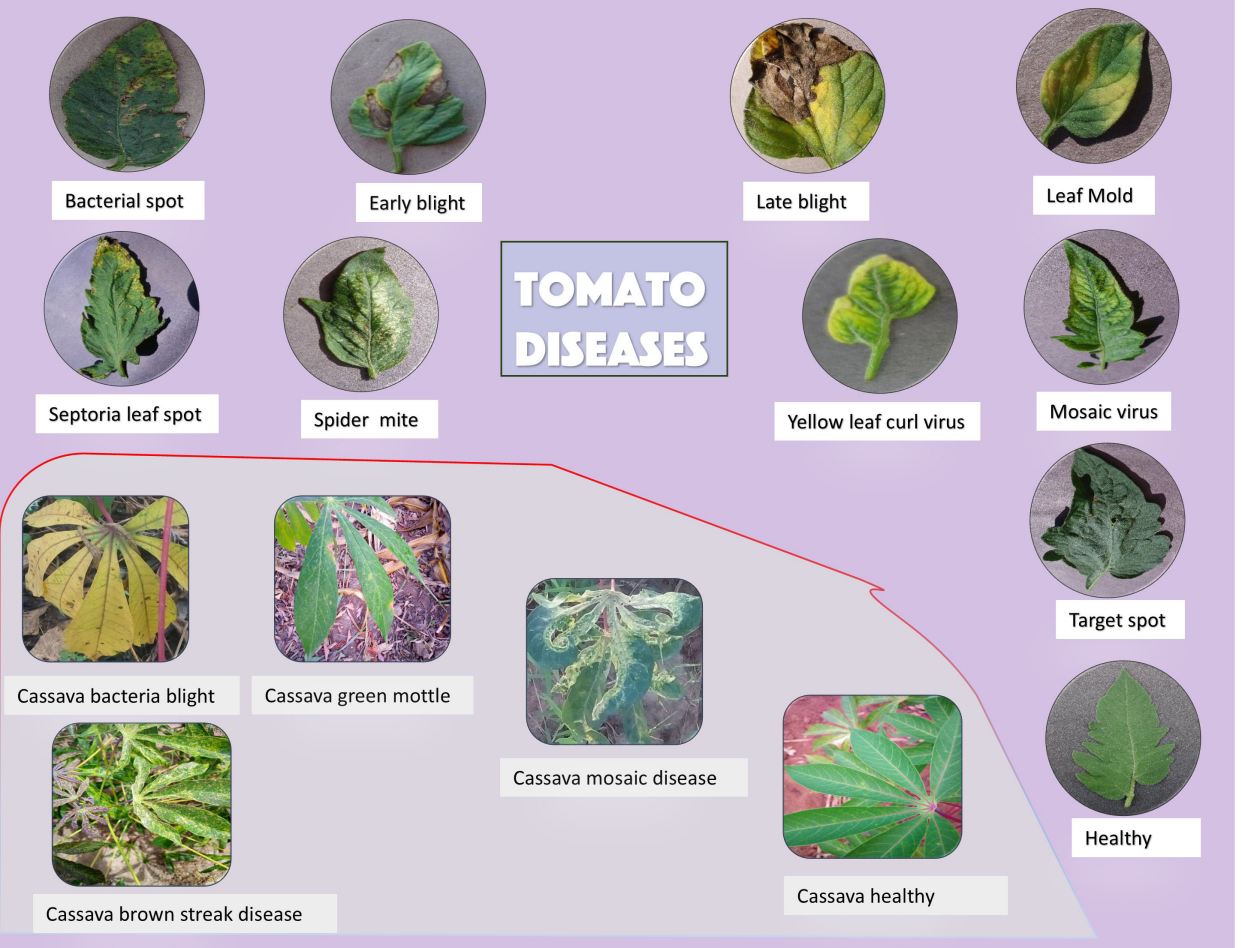
Collage of images showing tomato and cassava leaf diseases with labels. Tomato diseases include bacterial spot, early blight, late blight, leaf mold, septoria leaf spot, spider mite, yellow leaf curl virus, mosaic virus, and target spot. A healthy tomato leaf is also shown. Cassava diseases include bacteria blight, green mottle, mosaic disease, and brown streak disease, alongside a healthy cassava leaf. The background is light purple.

To evaluate the performance of the proposed method in this study, analysis was conducted using two datasets: the cassava leaf disease dataset and the PlantVillage repository (Hughes et al., 2015), which contains 18162 images of tomato leaf diseases. The cassava leaf disease dataset, updated by Gomez-Pupo et al (Gómez-Pupo et al., 2022), consists of 6,745 images, with 80% allocated for training, 10% for validation, and 10% for testing, respectively. **Figure 1** shows the different cassava and tomato leaf diseases. The tomato leaf disease dataset comprised 10 distinct classes, including nine disease classes and one healthy class. In both analyses, all images were resized to 224 x 224 for experimental purpose.

### 2.2 Networks

Three distinct networks ResNeT (RNet), Xception, and EfficientNetb (EffNetb) were employed in this study. ResNet, also known as Residual Network (He et al., 2016), is a deep learning framework that utilizes residual connections or skip links to circumvent levels within the network. These connections enable the training of extremely deep networks by resolving the issue of disappearing gradients and enhancing the flow of gradients, resulting in improved performance across many tasks. Xception is a neural network architecture created by Francois Chollet et al (Chollet, 2017). It combines depthwise separable convolutions with pointwise convolutions to achieve deep learning. This architecture improves image identification performance by replacing traditional Inception modules with depthwise separable convolutions, resulting in 36 convolutional layers and linear residual connections. EffNetb (Tan and Le, 2019) is a convolutional neural network. Architecture is known for its efficiency in terms of accuracy and computational resources. In 2019, Google AI researchers developed EfNetb. The core idea behind EfficientNet is to balance model width, depth, and resolution in order to improve performance without significantly increasing computational costs.

### 2.3 Preliminaries

Let $x\;\in \;R^{W\times H\times C}$ and *y* represent the training images and their labels, respectively. W and H, the width and height of an input image. (*y*_1_ and *y*_2_) represent the source and the target labels, $x_{1}\;\in \;R^{W\times H\times C}$ and $x_{2}\;\in \;R^{W\times H\times C}$ represent the source and the target image.

### 2.4 Algorithm of Enhanced-RICAP

**Figure 2:**
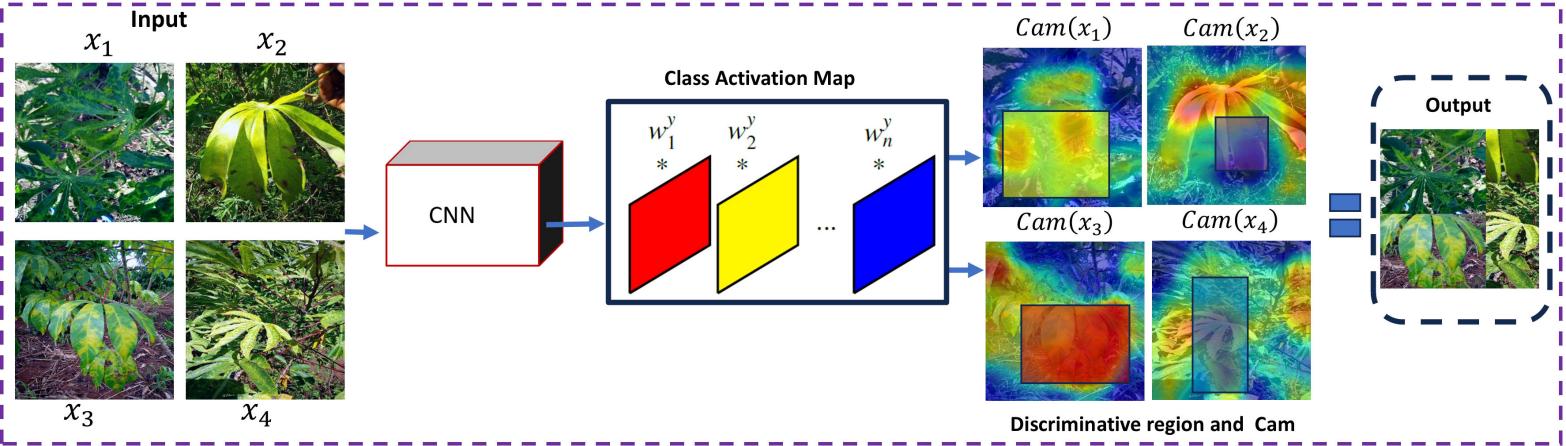
Diagram showing a process involving cassava leaves analyzed by a Convolutional Neural Network (CNN). Input images of leaves are processed through the CNN, generating class activation maps highlighting discriminative regions. The maps are then used to form an output collage, illustrating the areas of interest in the original images.


The main purposal of Enhanced-RICAP is to generate new training samples ( $\bar{x},\bar{y}$ ) to increase data diversity, whereby label noise is mitigated. Inspired by RICAP, which crops randomly four patches from four distinct images and patches them together from the upper left to the bottom right to generate augmented images. In RICAP, it has been observed that the randomly generated patches may cover meaningless information about the source image; therefore, mixing label proportionally to area-based static may lead to label noise and mislead the training process. To overcome the aforementioned limitation, we introduced a new data mixing augmentation technique named Enhanced-RICAP, specially designed for plant disease identification. **Figure 2** provides a comprehensive overview of the proposed Enhanced-RICAP method. The random region generation module in RICAP is replaced by the attention region generation module in Enhanced-RICAP. In each iteration, Enhanced-RICAP leverages the class activation map to obtain discriminative regions *P*_1_ *,…,P*_4_ of four distinct images *x*_1_ *,…,x*_4_ respectively, as in Algorithm 1. This process is accomplished in **Algorithm 1**. Subsequently, **Algorithm 1** is incorporated into **Algorithm 2** to complete the training. The class activation map is obtained from the last convolutional layer of the network and can be expressed as shown in Equation 1:

(1) $$Cam(x_{i})=\sum_{l=0}^{d}w_{y_{i}}^{l}F_{l}(x_{i}),$$

Algorithm 1 **Enhanced-RICAP at the beginning of the algorithm section.**


1 **Input:** a CNN function *f*, a training images (*x*
_1_,…*x_i_
*), where *I* is the images and *L* is the labels.
2 **for** *k in range (4)* **do**
3   *P_i_
* ← obtain the discriminative region of *x_i_
* with Equations 4, 5
4   
$\bar{x}$
 ← paste *P_i_
* into the corner left of *x*
_1_
5   else paste it according to the previous paste patches.
6 **end**

Algorithm 2 **Augmented Samples at the beginning of the algorithm section.**

**1 Input:** a CNN function *f*, a training sample (*I*, *L*), where *I* denotes the images and *L* denotes the labels.
2 **for** *epoch in range (epochs)* **do**
3 **for** (*I*, *L*) *in training samples* **do**
4 *r_ex_
* = randomly shuffle the batch images;
5 
$\bar{x}$
 ← generate the new training image using Algorithm 1
6 
$\bar{y}$
 ← generate the new training label using Equations 6-7
7 doning backpropagation to optimize *f* using the new training samples
8 **end**
9 **end**

where $F\;(x_{i})\;\in \;R^{d\times h\times w}$ signifies the result of the final convolutional layer, $F_{l}\;(x_{i})\;\in \;R^{h\times w}$ represents the *l^th^* feature map of *F*(*x_i_*) and $\omega_{y_{i}}^{l}\;\in \;R^{d}$ represents the weight in the fully connected layer associated with class *y_i_*. The most salient regions coordinates ${\bar{u}}^{x_{i}},{\bar{v}}^{x_{i}}$ can be obtained as shown in the Equation 2:


(2) $$\begin{array}{c} {\bar{u}}^{x_{i}},{\bar{v}}^{x_{i}}=\underset{{\bar{u}}^{x_{i}},{\bar{v}}^{x_{i}}}{\text{argmax }\;}(C\bar{a}m_{i}^{{\bar{u}}^{x_{i}},{\bar{v}}^{x_{i}}}) \end{array}$$

Since the coordinates above are in the class activation dimension, we use the Equation 3 below to convert the coordinates back to the original image dimension as:


(3) $$\begin{array}{c} u^{x_{i}}={\bar{u}}^{x_{i}}\times \frac{W}{w},\;v^{x_{i}}={\bar{v}}^{x_{i}}\times \frac{H}{h} \end{array}$$

Where *w* and *h* are the width and height of the class activation map of *x_i_*. The parameter *γ_i_* is used to define the width and height of the discriminative region *P_i_* of image *x_i_* by *w_i_* = *W × γ_i_*, and *h_i_* = *H × γ_i_*. The follow Equation expresses how to extract the discriminative region *P_i_* of *x_i_* as:


(4) $$\begin{array}{ll} u_{l}^{x_{i}} & =u^{x_{i}}-\frac{w_{i}}{2},\;u_{r}^{x_{i}}=u^{x_{i}}+\frac{w_{i}}{2}\\ v_{t}^{x_{i}} & =v^{x_{i}}-\frac{h_{i}}{2},\;v_{b}^{x_{i}}=v^{x_{i}}+\frac{h_{i}}{2} \end{array}$$

(5) $$\begin{array}{ll} \text{if }u_{l}^{x_{i}}\leq 0,\{\begin{array}{l} u_{l}^{x_{i}}=0\\ u_{r}^{x_{i}}=w_{i} \end{array} & \text{if }u_{r}^{x_{i}}\geq W,\{\begin{array}{l} u_{l}^{x_{i}}=W-w_{i}\\ u_{r}^{x_{i}}=W \end{array}\\ \text{if }v_{b}^{x_{i}}\leq 0,\{\begin{array}{l} v_{b}^{x_{i}}=0\\ v_{t}^{x_{i}}=h_{i} \end{array} & \text{if }v_{t}^{x_{i}}\geq H,\{\begin{array}{l} v_{b}^{x_{i}}=H-h_{i}\\ v_{t}^{x_{i}}=H \end{array} \end{array}$$

Where $P_{i}(u_{l}^{x_{i}},u_{r}^{x_{i}},v_{b}^{x_{i}},v_{t}^{x_{i}})$ to denote the discriminative region of the image *x_i_*, and $u_{l}^{x_{i}},u_{r}^{x_{i}},v_{b}^{x_{i}},v_{t}^{x_{i}}$ represent the left, right, bottom, and top boundaries of the region *P_i_*. At the end, those discriminative regions $P=\{P_{1},\ldots ,P_{4}\}$ are patched together the upper left, upper right, lower left, and lower right regions to generate the augmented image.

#### 2.4.1 Label mixing

In RICAP, the mixed labels are computed based on the proportion of the image area that comes from each source image. It is observed that area-based labels mixing may not reflect the intrinsic composition of the mixed images, which can cause model instability. To tackle this issue, we exploit the class activation map of each to obtain the intrinsic semantic composition of each region that composed the mixed image. This operation can be expressed as follows:


(6) $$\begin{array}{l} \lambda_{i}=\sum \text{Φ}(Cam(P_{i})/\text{Φ}(Cam(x_{i}))) \end{array}$$

where Φ denotes the operation that enlarges the dimensions of a feature map to align with those of the image *x_i_*. The notation $\bar{y}$ represents the target label vector corresponding to the four mixed images. It is computed by multiplying each original one-hot class label vector *y_i_* by its associated label weight *λ_i_*, which reflects the image’s contribution to the augmented sample, and summing the four weighted vectors for *i* = 1*,…*,4.


(7) $$\bar{y}=(\underset{i\in \{1,2,3,4\}}{\sum }{\text{λ}}_{i}y_{i})$$

## 3 Results

### 3.1 Ablation study outcomes for the proposed method on Cassava leaf diseases

In our analysis, the standard deviation of the values is denoted by the numbers following the *±* operator, each method was computed over four distinct runs. Consequently, in this section, we performed tests to compare our proposed method with previously existing studies utilizing RNet18, RNet34, RNet50, EffNetb0, and Xception with pretrained ImageNet weights. Considering that these studies did not officially report results on cassava leaf disease and tomato leaf disease, the methods were implemented based on the released codes and conducted experiments on the two datasets. We initially explored various hyperparameters for each method and identified the optimal one for the network architecture. We defined the hyperparameters as 0.5 for Cutout and CutMix, and 1.0 for MixUp, and used alpha values of 1.0 and 3.0 for CutMix and MixUp, respectively. An initial learning rate of 0.0001 and a weight decay of 1e-5 were applied using the Adam optimizer. Compared to the SGD optimizer, Adam has been observed to achieve higher accuracy on the cassava leaf disease dataset. The comparative analysis of test accuracy is presented in **Table 1**. The proposed method is evaluated against state-of-the-art techniques. When using the RNet18 network, Enhanced-RICAP demonstrates superior performance, exceeding MixUp by 0.83% and Hide and Seek by 0.51%. Similarly, with RNet34, Enhanced-RICAP outperforms CutOut by 2.16% and MixUp by 1.36%. However, the performance of the proposed method using RNet50 is comparatively lower than that achieved with RNet34. Furthermore, when applied to EffNetb0, the method achieves a marginal improvement of 0.2% over ResizeMix. Furthermore, when applied to EffNetb0, the method achieves a marginal improvement of 0.2% over ResizeMix. Notably, it significantly surpasses Hide and Seek and CutMix by 1.55% and 2.51%, respectively, when integrated with the Xception architecture The experimental results, as presented in **Table 2**, reveal that our methodology achieved superior performance compared to RICAP and ResizeMix techniques when evaluating test error rates using Xception on the cassava leaf disease identification (CLDD). Furthermore, our approach substantially surpassed the baseline, underscoring the consistent effectiveness of Enhanced RICAP across diverse network architectures.

**Table 1:** Comparison of different methods and their accuracy on cassava leaf disease dataset.

Method	Accuracy (%)			
	RNet18	RNet34	RNet50	EffNetb0
Baseline	91.18 *±* 0.70	93.58 *±* 1.03	92.46 *±* 0.48	91.66 *±* 0.56
CutMix	91.02 *±* 0.99	91.50 *±* 0.054	92.62 *±* 0.21	89.58 *±* 0.31
MixUp	91.18 *±* 0.07	92.78 *±* 0.38	91.02 *±* 0.30	90.54 *±* 0.26
ResizeMix	90.54 *±* 0.019	91.98 *±* 0.26	71.15 *±* 0.18	93.58 *±* 0.33
CutOut	90.70 *±* 0.65	91.98 *±* 0.41	92.42 *±* 0.85	91.02 *±* 0.22
Hide and Seek	91.50 *±* 0.33	91.66 *±* 0.19	91.67 *±* 0.28	89.74 *±* 0.011
Enhanced-Ricap	92.01 *±* 0.18	94.14 *±* 0.22	93.18 *±* 0.031	93.78 *±* 0.26

**Table 2:** Top-1 error rates comparison of ResizeMix, RICAP, and our method on cassava leaf disease.

Model + Method	epochs	Top-1 Err (%)
Baseline	200	8.50 *±* 1.250
Baseline+ResizeMix	200	9.46 *±* 0.20
Baseline+Ricap	200	6.88 *±* 0.83
Baseline+Enhanced-Ricap	200	3.36 *±* 0.43

### 3.2 Evaluation of Xception-based cassava leaf disease identification using confusion matrix analysis

**Figure 3:**
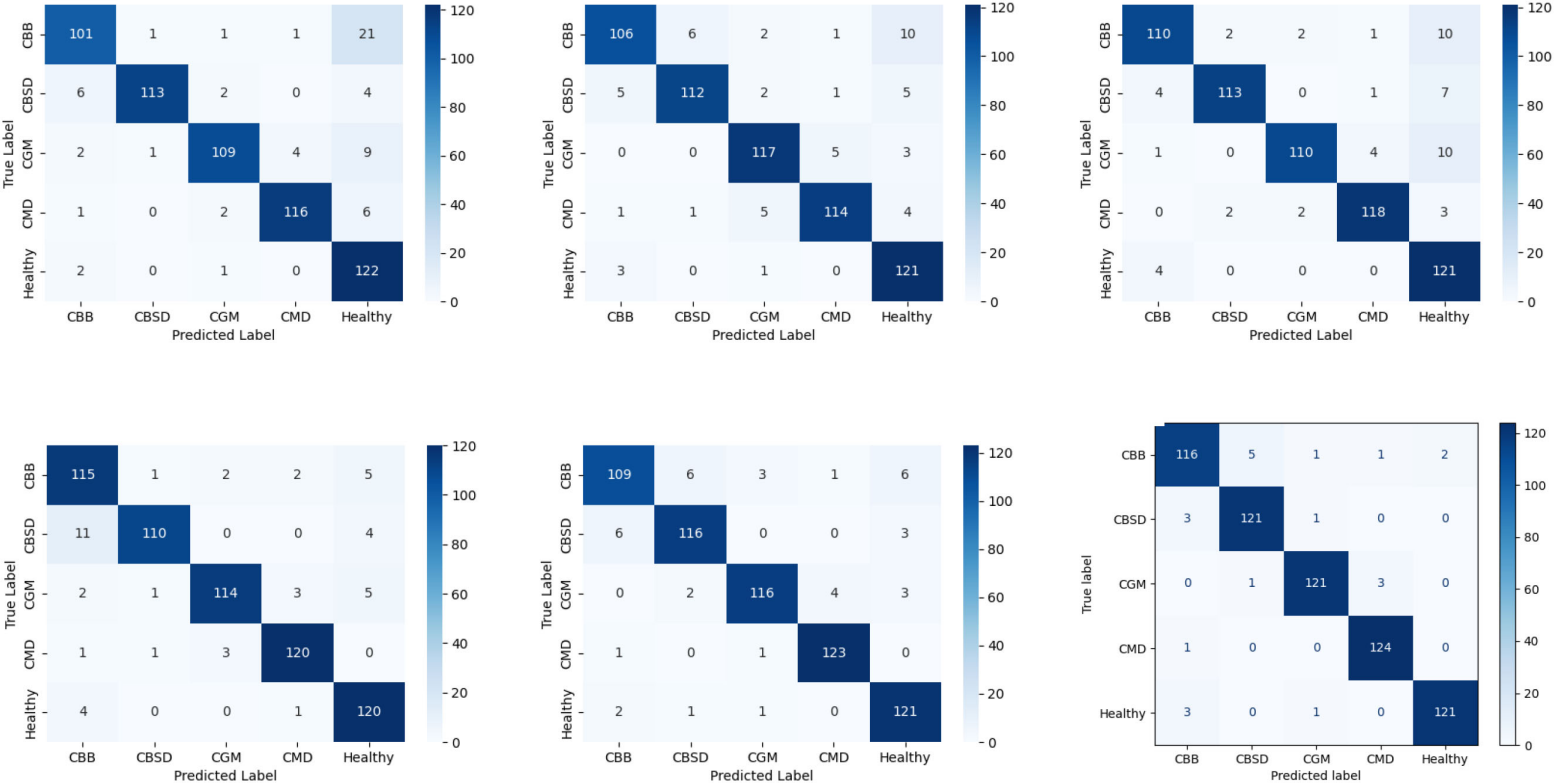
Six confusion matrices display classification results for five categories: CBB, CBSD, CGM, CMD, and Healthy. Each matrix varies slightly, with dark blue squares indicating higher accuracy. The matrices compare true labels versus predicted labels, using a color gradient from light to dark blue, representing values from zero to one hundred twenty.

By using Xception with the suggested approach, 625 untrained images, comprising four categories of cassava leaf diseases and healthy leaves, were chosen for identification. The resulting confusion matrix for recognizing cassava leaf diseases is depicted in **Figure 3**. The blue background illustrates identification accuracy, with a darker blue color indicating a higher level of identification accuracy. The confusion matrix reveals that our method achieves the highest identification accuracy, with 603 images correctly identified when distinguishing between four main cassava leaf diseases and healthy leaves. Among these, CBB exhibits the highest error rate, 5 of the 9 images that were incorrectly recognized were classified as CBSD. Therefore, there were mutual identification errors between CBB and CBSD. Bacterial blight and brown streak disease both cause yellowing of leaves. Similarly, errors in disease identification occurred because the spots associated with various diseases appeared alike at the same time. In addition, the numbers of correct identifications were 561 for Resizemix, 570 for CutOut, 572 for CutMix, 579 for Hide and Seek, and 585 for MixUp. The confusion matrix was utilized to calculate the accuracy, recall, precision, and F1-score for the five cassava leaf categories, serving as performance evaluation indicators for our method, as presented in **Table 3**. The proposed method achieves an average accuracy of 96.64% in classifying four types of cassava leaf disease along with healthy leaf images. Additionally, the method attains an average precision of 96.4%, an average F1-score of 96.4%, and an average recall of 96.6%. These results demonstrate the effectiveness of the approach in accurately recognizing cassava leaf.

**Table 3:** Comparative analysis of precision, recall, and F1-score on cassava leaf disease.

Categories	Precision (%)	Recall (%)	F1-score (%)
CBB	0.98	0.90	0.94
CBSD	0.98	0.98	0.98
CGM	0.96	0.98	0.97
CMD	0.95	0.99	0.97
Healthy	0.95	0.98	0.96

### 3.3 Evaluation

As shown in **Table 4**, a comprehensive comparison of various training methods and their corresponding accuracy on the cassava leaf disease dataset when training from scratch, without utilizing ImageNet pre-trained weights. This analysis demonstrates that our proposed method remains effective even in the absence of pre-trained weights, highlighting its adaptability. it is evident that the baseline model using RNet34 achieved an accuracy of 84.45%, which is higher than the accuracies obtained by the baselines on other Convolutional Neural Networks. Specifically, the baseline models using RNet18, RNet50, EffNetb0, and Xception achieved accuracies of 83.17%, 81.21%, 78.43%, and 82.60%, respectively. This indicates that RNet34 may be particularly well-suited for this dataset when trained from scratch. Among the various augmentation techniques applied, Xception with CutOut exhibited the lowest identification accuracy of 72.27%. In contrast, CutMix, MixUp, Hide and Seek, and Enhanced-RICAP achieved identification accuracies ranging between 80% and 90%. Notably, Enhanced-RICAP demonstrated the highest accuracy of 90.01%, surpassing all other methods. This highlights the efficacy of Enhanced+Ricap in improving model performance through advanced data augmentation and training strategies.

**Table 4:** Performance analysis of state-of-the-art models on cassava leaf disease identification without pre-trained weights.

Method	Accuracy (%)				
	RNet18	RNet34	RNet50	EffNetb0	Xception
Baseline	83.17 + 0.12	83.58 + 0.042	81.21 ± 0.15	78.43 ± 0.85	82.60 + 0.60
CutMix	83.17 ± 0.40	84.45 ± 0.77	83.65 + 0.26	85.89 + 0.13	79.01 + 0.56
MixUp	83.97 + 0.67	82.32 ± 0.27	83.01 ± 0.053	85.89 + 0.15	76.76 + 0.81
CutOut	82.53 ± 0.94	84.45 + 0.065	83.17 + 0.35	86.21 + 0.73	72.27 + 0.38
Hide and Seek	84.29 + 0.36	83.49 + 0.012	84.29 ± 0.041	89.58 ± 0.23	73.07 ± 0.47
Enhanced+Ricap	84.43 + 0.024	84.75 ± 0.17	84.82 ± 0.04	90.01 + 0.083	83.10 + 0.052

### 3.4 Analysis of experimental results on a publicly available tomato leaf disease dataset

**Table 5** presents a comprehensive comparison between the proposed technique and previous studies, utilizing classical models such as RNeT18 as common baselines with our method. The results consistently indicate that Enhanced-Ricap outperforms the models proposed in these studies by a considerable margin. When comparing the Enhanced-Ricap to other recent methodologies, its superiority becomes increasingly apparent. For example, Li et al. (2023) documented an accuracy rate of 99.70%, whereas Enhanced-Ricap demonstrated an accuracy of 99.86%. This indicates that our proposed method exhibits a 0.16% enhancement in accuracy relative to the findings. Similarly, Paul et al. (2023) reported an accuracy of 89.00%, reflecting a 2% improvement over VGG16, which was significantly lower than the 12.87% enhancement demonstrated by our method. Moreover, although Sanida et al., 2023 reported notable improvements with RNeT50 and VGG16, yielding gains of 2.33% and 2%, respectively, these gains remained inferior to those achieved by Enhanced-Ricap. In comparison, Zarboubi et al. (2025) reported an accuracy of 99.12%, with a 1.22% improvement over RNeT50, while Enhanced-Ricap delivered 99.86% accuracy, further underscoring its superior performance. These comparisons highlight the capability of the model to demonstrate enhanced metrics in various evaluation criteria, including precision, recall, F1-score and accuracy, thereby solidifying its position as a leading solution for complex identification tasks.

**Table 5:** Comparative performance analysis of existing methods and our approach.

Authors	Models+Method	Accuracy	Precision	Recal	F1-Score
Li et al. (2023)	Custom-LMBRNet	99.70	99.72	99.66	99.69
Yang et al. (2024)	Custom-LSGNET	95.54	93.62	94.13	93.78
Paul et al. (2023)	Custom-CNN	89.00	89.00	89.00	89.00
Sanida et al. (2023)	Custom-CNN	99.63	99.12	99.29	99.20
Zarboubi et al. (2025)	Custom-CNN	99.12	99.13	99.12	99.11
Our	RNet18+Enhanced-Ricap	99.86	99.76	99.69	99.73

### 3.5 Application and deployment of a mobile app for plant disease identification

**Figure 4:**
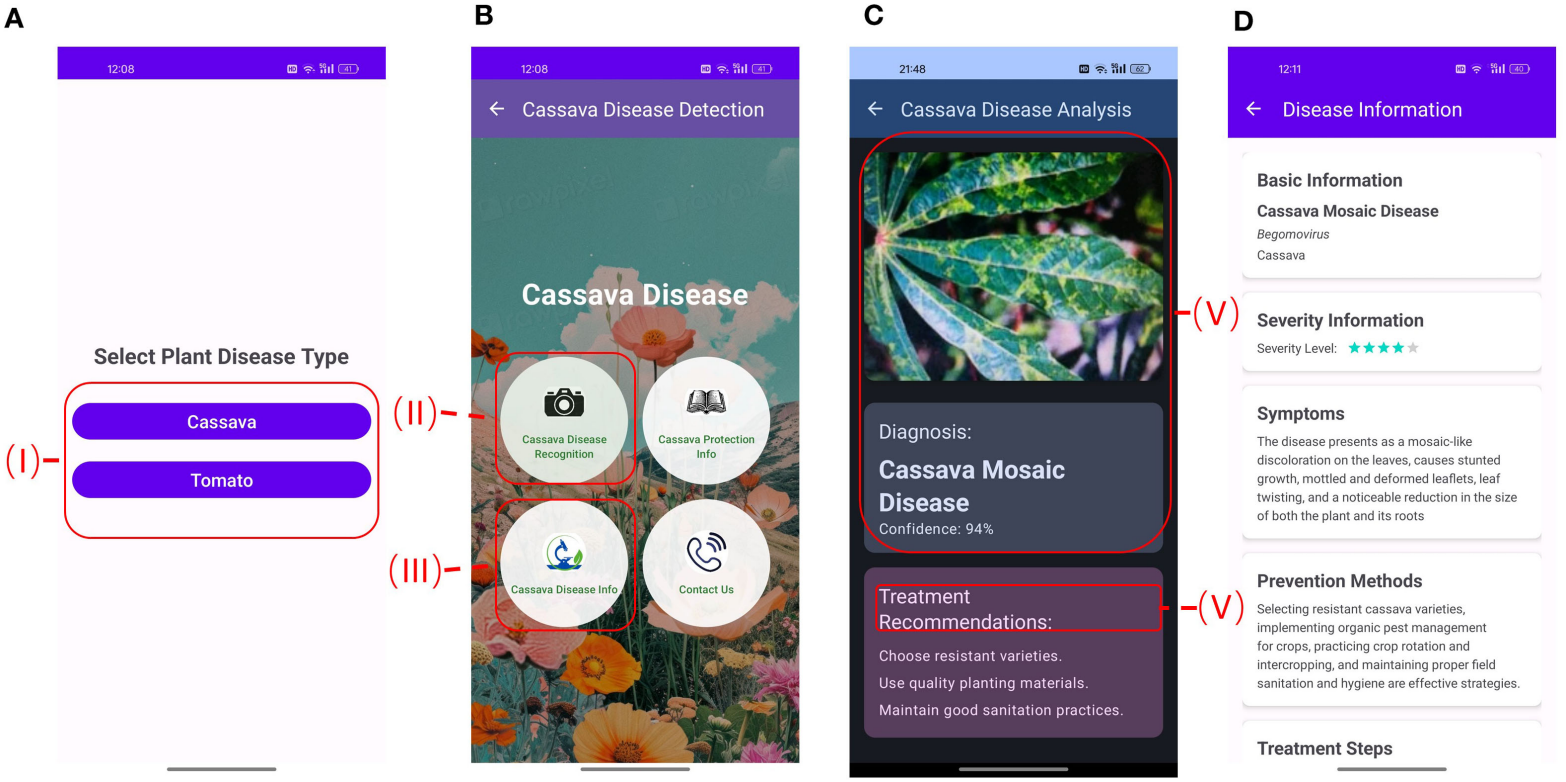
A series of mobile app screens for plant disease detection. Panel A shows a selection screen for cassava or tomato plant diseases. Panel B displays options for cassava disease recognition, protection, information, and contact. Panel C provides a diagnosis of cassava mosaic disease with a confidence level of 94 percent and treatment recommendations. Panel D outlines disease information, including basic information, severity level (three stars), symptoms, and prevention methods for cassava mosaic disease, detailing management strategies.

This section focuses on the selection of an optimal model, such as RNeT18 with Enhanced-RICAP, designed to function efficiently on mobile devices while minimizing computational cost. To improve accessibility and practical utility for supporting farmers and agricultural experts, we developed a mobile app using Android Studio. The model is embedded within the application and executes locally on the device, thereby facilitating disease identification without reliance on an internet connection. The user interface of the PlantDisease Android application is depicted in **Figure 4**. The home screen of the PlantDisease include two primary options that can be select between Tomato and Cassava in (I in **Figure 4A**). To start an identification request, users can click the camera icon, which provides alternative options to either capture a new image or by uploading an existing image, as shown (II in **Figure 4B**). After the system finishes identifying, the result is delivered to the Disease Analysis screen (in **Figure 4C**). If a diseases is detected the predicted image, the diagnosed disease, the prediction confidence score (IV in **Figure 4C**), and a brief treatment recommendation (V in **Figure 4C**), Along with corresponding information about possible diseases, their symptoms, methods, and treatments steps are provided (**Figure 4D**). Function panel (I in **Figure 4A**) can be requested by tapping the respective buttons in Cassava or Tomato Disease Info (III in **Figure 4B**). The function panel provides access to other tools (I in **Figure 4A**): the book icon links to button of tomato disease general prevention measures, the plant leaf icon links to an introduction of the nine tomato diseases assessed in this work, and the phone icon links to the contact information of the PlantDisease development team.

### 3.6 Class activation mapping visualization

The visualization of CAM heatmaps was conducted using the following techniques: the baseline.

**Figure 5:**
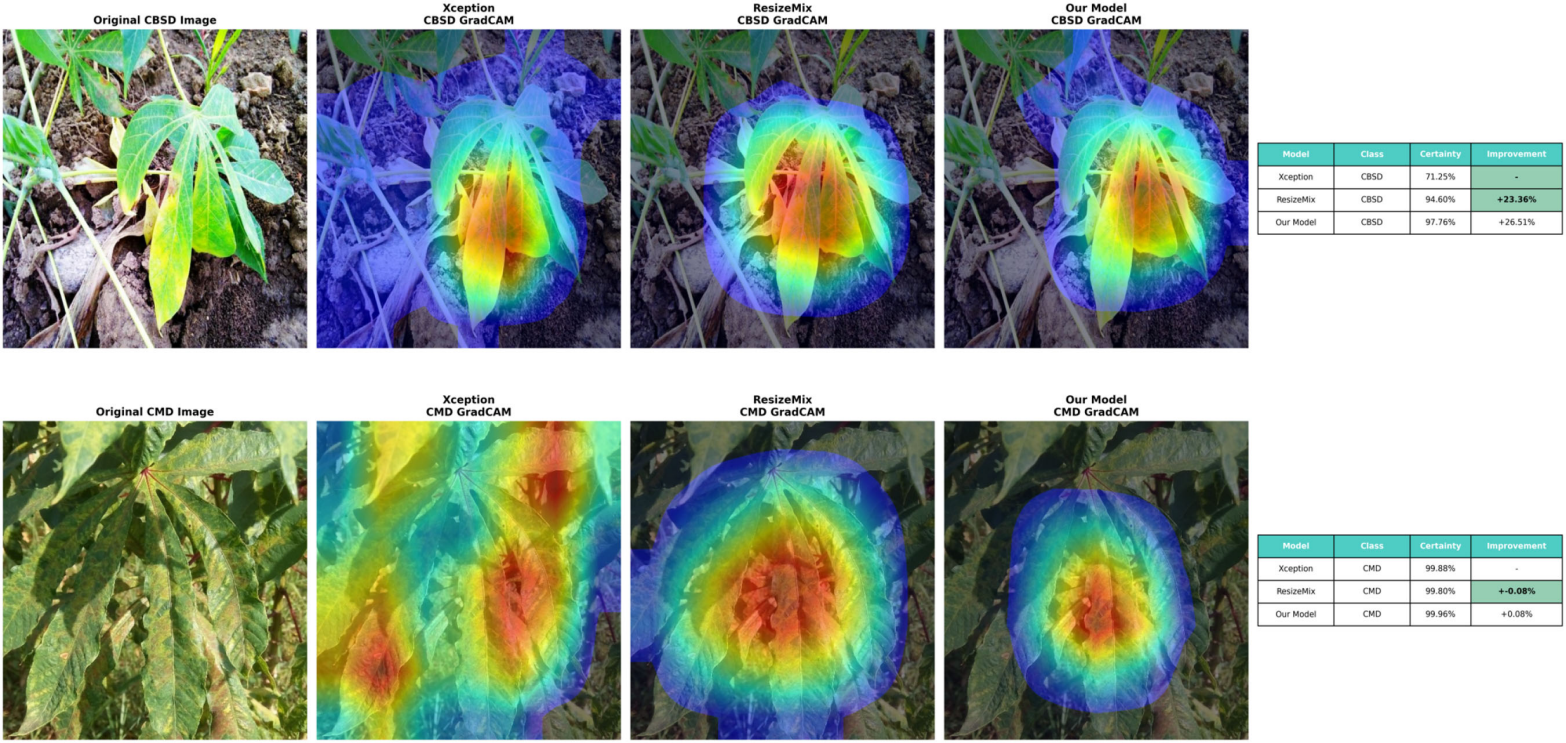
Comparison of cassava leaf disease detection methods. The top row shows an original CBSD image, Xception Grad-CAM, ResizeMix Grad-CAM, and a model's Grad-CAM. The table displays certainty and improvement percentages for CBSD detection. The bottom row shows an original CMD image with similar Grad-CAM visualizations. Another table shows certainty and improvement for CMD detection. The model demonstrates notable improvements over others.

Xception model, ResizeMix, and our proposed method. Specifically, during visualization, the attention heatmap was merged with the original image, as shown in **Figure 5**. This approach allows a direct comparison between the original image and the outputs of the baseline, ResizeMix, and our method. Notably, darker colors in the heatmap correspond to higher activation values, highlighting regions most relevant for decision-making. Furthermore, compared with the baseline model and ResizeMix, our method demonstrates stronger feature extraction and improved detection of Cassava leaf disease, effectively capturing diverse color patterns and contextual information. In particular, Enhanced-RICAP further directs the network’s attention to the most informative object regions, emphasizing discriminative features while reducing sensitivity to background noise. Consequently, these results indicate that Xception and ResizeMix can struggle with accurately discriminating leaf colors and extracting relevant background information. In contrast, the proposed method shows superior understanding of sample features and more effectively identifies key regions for classification.

## 4 Discussion

In this current study, different models and methods were employed, all aiming to achieve high accuracy. Maryum et al. (2021) used EffNetb4 with an 85:15 train-validation split, achieving 89.09% accuracy. Chen et al. (2022) used RNet50 in a fivefold cross-validation, improving accuracy to 89.7%. Vijayalata et al. (2022) achieved 92.6% accuracy with EffNetb0 using an 80:20 split, while Thai et al. (2021) used Vision Transformer with the same split, reaching 90.0%. Methil et al. (2021) also used EffNetb4 with an 80:20 split, obtaining 85.64% accuracy. The proposed model in this current study, combining Enhanced-RICAP with transfer learning using the Xception model, outperformed all others with an accuracy of 96.64%, demonstrating the effectiveness of this approach for cassava leaf disease identification. Our findings are consistent with previous studies that utilized the weights of the MobileNetV2 CNN model to classify cassava images, leveraging the extensive visual knowledge acquired from the ImageNet database (Tewari and Kumari, 2024). In addition, Karpathy et al. (2014) demonstrated that transfer learning is effective across various applications and significantly reduces computational demands compared to training from scratch, which is advantageous for machine applications.

In this current study, a comprehensive overview of identification performance for cassava disease identification demonstrates robust results across all categories. The precision, Recall, and F1 scores for each disease category highlight the effectiveness of the model. CBB and CBSD achieved high scores, with CBSD slightly outperforming CBB in all metrics. Notably, CGM and CMD showed exceptional performance, with CMD achieving the highest scores across all metrics, indicating particularly effective identification. Previous studies have underscored the importance of precision and Recall in agricultural disease identification, often noting trade-offs between these metrics. Research by Mohanty et al. (2016) and others has demonstrated that high precision and Recall are crucial for practical applications in disease identification. The high scores for CGM and CMD in this study align with findings from similar works, which suggest that advanced models and techniques can lead to more accurate and reliable identification. The model’s overall accuracy of 0.96.64, with a macro-average precision, Recall, and F1-score of 0.96, reflects its robustness and reliability, consistent with recent advancements in deep learning for agricultural applications. This performance reinforces the findings of previous research, which highlights the efficacy of state-of-the-art models in achieving high identification accuracy across diverse classes. The strong results across all classes, including the Healthy class, further demonstrate the model capability to distinguish between the leaves of diseased and healthy cassava plants effectively, supporting its practical utility in real-world scenarios.

Previously, Jiang et al. (2021) reported that Class Activation Maps are designed to highlight the regions in an image that a convolutional neural network (CNN) considers most relevant for identifying a specific category. This approach leverages the spatial information present in each activation map, with convolutional layers closer to the network’s identification stage providing more meaningful high-level activations. These activations are used for visual localization, helping to explain the network’s final prediction. Previous research highlights the importance of CAM in improving model transparency and understanding. Techniques such as Grad-CAM and its variants have been shown to enhance interpretability by providing visual insights into which parts of an image contribute to the final identification (Selvaraju et al., 2020). The findings from this study provide essential information about how CAM have developed over the years with the establishment of robust evaluation metrics and the development of relatively high-performing models such as Enhanced-RICAP, the future trajectory of CAM-based methods shifting toward more application-oriented research. Our findings align with these observations, demonstrating that CAM can effectively highlight the strengths of the proposed model and reveal areas where other augmentation techniques may fall short. This aligns with the broader understanding that CAM visualization is crucial for validating model performance and ensuring that the decision-making process is both accurate and interpretable.

Crop diseases continue to impose substantial financial burdens on farmers, significantly reducing yield and compromising both food quality and environmental health. The lack of access to advanced diagnostic technologies often results in ineffective disease management, leading to soil degradation, increased chemical use, and disruptions in the food supply chain. Traditional methods, while informative, are often time-consuming, subjective, and limited in scalability. In response to these challenges, we developed a deep learning-based system, PlantDisease, utilizing an enhanced Xception+Enhanced-RICAP architecture fine-tuned for precision and efficiency. Our model was trained on annotated dataset encompassing four (Alford and Tuba, 2024) cassava diseases and nine (Ferentinos, 2018) tomato diseases. Integrated into a user-friendly Android application, the system not only classifies diseases with 96.64% accuracy, but also provides users with detailed information on symptoms, prevention strategies, and recommended treatment protocols. Compared to prior studies, our results demonstrate improved performance. For instance, Mohanty et al. (2016) achieved 91.2% accuracy in classifying 26 diseases across 14 crop species using AlexNet and GoogLeNet models. Similarly, Ferentinos (2018) reported an average accuracy of 99.53% using deep convolutional neural networks across 58 disease classes, but with limitations in mobile deployment and real-time feedback. Unlike these studies, our model prioritizes both accuracy and real-world usability through a lightweight architecture optimized for mobile platforms. Furthermore, our approach improves upon the generalizability and interpretability challenges observed in earlier works by incorporating disease-specific guidance and interactive support within the application. The inclusion of contextual knowledge such as visual symptoms and actionable management steps bridges the gap between automated identification and practical field application. In summary, our system not only advances the technical accuracy of plant disease identification but also enhances its accessibility and utility for farmers, contributing to more resilient agricultural systems and sustainable food production.

## 5 Conclusions

In this study, we introduced Enhanced-RICAP, a novel data augmentation method designed to enhance image identification accuracy while effectively mitigating model overfitting. We also developed PlantDisease, a mobile application developed specifically for the real-time identification of cassava and tomato leaf diseases. The method introduced in this study was rigorously evaluated using various benchmark deep learning architectures RNeT18, RNeT34, RNeT50, and Xception under identical training conditions. We compared Enhanced-RICAP against established augmentation techniques such as CutMix, MixUp, CutOut, Hide-and-Seek, and RICAP. Experimental results consistently demonstrated the superior performance of Enhanced-RICAP across key evaluation metrics, including accuracy, precision, recall, and F1-score. Notably, the RNeT18+Enhanced-RICAP configuration achieved an impressive accuracy of 99.86%, while preserving computational efficiency due to its lightweight architecture. Furthermore, the Xception+Enhanced-RICAP model attained 96.64% accuracy in classifying four cassava leaf disease, demonstrating the robustness of our approach across different model types and dataset. To ensure practical applicability, we integrated the RNeT18+Enhanced-RICAP model into the PlantDisease mobile app. This user-friendly application will empower farmers and agricultural practitioners to diagnose tomato and cassava leaf conditions promptly and accurately. In addition to disease identification, the application will provide straightforward recommendations for prevention and treatment. By enabling early and accurate diagnosis, PlantDisease will reduce the overuse or misuse of pesticides and lessens the dependency on expert intervention, thereby supporting sustainable agricultural practices. In this work, the effectiveness of the proposed method was evaluated using only two datasets. Nevertheless, in future research, the method can be extended to a broader range of plant disease identification and severity estimation dataset, encompassing diverse leaf images from various plants affected by different diseases. This expansion would not only test the method’s generalization and robustness across multiple plant species but also enhance its scalability and practical applicability.

## Data Availability

The original contributions presented in the study are included in the article/supplementary material. Further inquiries can be directed to the corresponding author.
